# Mucosal Immunization with Integrase-Defective Lentiviral Vectors Protects against Influenza Virus Challenge in Mice

**DOI:** 10.1371/journal.pone.0097270

**Published:** 2014-05-13

**Authors:** Judith M. Fontana, Paul J. Christos, Zuleika Michelini, Donatella Negri, Andrea Cara, Mirella Salvatore

**Affiliations:** 1 Department of Public Health, Weill Medical College of Cornell University, New York, New York, United States of America; 2 Department of Medicine, Weill Medical College of Cornell University, New York, New York, United States of America; 3 Department of Therapeutic Research and Medicines Evaluation, Istituto Superiore di Sanità, Rome, Italy; 4 Department of Infectious, Parasitic and Immune-mediated Diseases, Istituto Superiore di Sanità, Rome, Italy; University of Pittsburgh, United States of America

## Abstract

Recent reports highlight the potential for integrase-defective lentiviral vectors (IDLV) to be developed as vaccines due to their ability to elicit cell-mediated and humoral immune responses after intramuscular administration. Differently from their integrase-competent counterpart, whose utility for vaccine development is limited by the potential for insertional mutagenesis, IDLV possess a mutation in their integrase gene that prevents genomic integration. Instead, they are maintained as episomal DNA circles that retain the ability to stably express functional proteins. Despite their favorable profile, it is unknown whether IDLV elicit immune responses after intranasal administration, a route that could be advantageous in the case of infection with a respiratory agent. Using influenza as a model, we constructed IDLV expressing the influenza virus nucleoprotein (IDLV-NP), and tested their ability to generate NP-specific immune responses and protect from challenge *in vivo*. We found that administration of IDLV-NP elicited NP-specific T cell and antibody responses in BALB/c mice. Importantly, IDLV-NP was protective against homologous and heterosubtypic influenza virus challenge only when given by the intranasal route. This is the first report demonstrating that IDLV can induce protective immunity after intranasal administration, and suggests that IDLV may represent a promising vaccine platform against infectious agents.

## Introduction

Viral vectors represent an attractive platform for vaccine development due to their ability to effectively deliver genes of interest into cells, and generate humoral and cell-mediated immune responses [1]. Lentiviral vectors (LV) offer several specific advantages over other viral delivery systems because they can efficiently transduce slow-replicating and post-mitotic cells, including antigen-presenting cells, *in vivo*, while still allowing them to elicit robust, antigen-specific immune responses [2], [3], [4], [5]. As a safety measure, current generation LV are also replication-deficient, since all structural proteins required to construct the vector are supplied in *trans* to the packaging signal [6], and self-inactivating, due to a deletion in the 3′ long-terminal repeat region of the viral promoter and enhancer sequences [7]. Finally, preexisting immunity to LV is absent in humans, making them unlikely to be cleared by the host [8], a major hurdle for other vector-based strategies.

Despite their appealing features, integrase-competent LV (ICLV) are limited as vaccine delivery tools by their potential to integrate into host cell chromosomes [9], which poses the health risk of insertional mutagenesis. Integrase-defective LV (IDLV) share the favorable features of ICLV, but do not present this safety concern due to a mutation in the catalytic domain of the integrase (IN) protein that blocks integration [10], [11]. As a result, IDLV accumulate in the nuclei of transduced cells as stable, transcriptionally-active, episomal DNA circles [9], [12], [13] that persist in slowly dividing and terminally differentiated cells. From the perspective of vaccine development, both muscle (terminally differentiated cells) and airway epithelial cells (turnover >12 months)[14] represent ideal targets for IDLV administration because they would allow persistent antigen expression. In mice, IDLV circles were shown to be stable in the absence of integration, and transgene expression was present for at least 3 months (length of study) after administration in muscle [15]. Indeed, the intramuscular (i.m.) administration of IDLV expressing foreign antigens has been successfully exploited for vaccine development [16]. In this respect, the antibody response after i.m. administration of IDLV protected mice from lethal challenge with West Nile virus [4], and the T cell response to a human papillomavirus oncogenic protein expressed from i.m. IDLV was effective at eradicating established tumors in mice [17]. Recently, IDLV vaccination has also been shown to provide sterilizing immunity against malaria [18].

Although i.m. administration of IDLV has been shown to induce strong immune responses and protect from disease, there are no data regarding whether this result can also be achieved after inoculation via the intranasal (i.n.) route. The i.n. route of administration is often more effective than the i.m. route for inducing a protective immune response against pathogens that use the respiratory tract as their port of entry [19]. Given that IDLV effectively transduce and persist in quiescent cells, which make up ∼95% of the epithelial cell population in the airway, genetic vaccination using IDLV by the i.n. route would allow for persistent antigen expression and presentation in the airways, and may be ideal to elicit a protective response against infectious respiratory agents.

In this study, we evaluated the ability of IDLV to induce broad-based humoral and cell-mediated immunity, and most importantly, to protect from lethal challenge with an infectious respiratory agent. For the first time, we also compared the effectiveness of IDLV when administered by either the i.n. or i.m. route. To test these parameters, we chose influenza infection in BALB/c mice as model system. Influenza virus enters though the airways, and both humoral and cell-mediated immunity have been shown to contribute to protection against infection. We chose to express the internal nucleoprotein (NP) of influenza virus from IDLV (IDLV-NP) because NP is >90% conserved among influenza virus strains [20], and because it is the major target of the cross-protective T cell response against influenza virus in the mouse model [21], [22], [23], [24]. Antibodies elicited *in vivo* by NP may also help to accelerate virus clearance and promote resistance to influenza virus infection [25].

We found that IDLV-NP induces NP-specific T cell and antibody responses when administered *in vivo* by either the i.n. or i.m route. Protection from lethal influenza challenge was dependent on the route of administration, and IDLV-NP conferred protection against homologous and heterosubtypic strains of influenza virus only when given via the i.n. route. Taken as a whole, our data suggests that IDLV are a promising vaccine platform, and that this vector can also be effective when administered i.n.

## Materials and Methods

### Cells

Madin-Darby Canine Kidney (MDCK-ATL) Cells, FR-926, and Madin-Darby Canine Kidney (MDCK) Cells, London Line, FR-58, were obtained through the Influenza Reagent Resource, Influenza Division, WHO Collaborating Center for Surveillance, Epidemiology and Control of Influenza, Centers for Disease Control and Prevention, Atlanta, GA. The human embryonic kidney 293T cell line was purchased from American Type Culture Collection (ATCC, Manassas, VA), and the Lenti-X 293T cell line was obtained from Clontech (Mountain View, CA). Cells were maintained in Dulbecco's Modified Eagle's Medium (DMEM; Lonza, Allendale, NJ) containing 10% fetal bovine serum (FBS; Thermo Scientific, Waltham, MA), 100 IU/ml penicillin, and 100 µg/ml streptomycin. All cells were incubated at 37°C and 5% CO_2_. Influenza virus-infected cells were grown in DMEM containing 0.5% bovine albumin and TPCK trypsin (Sigma-Aldrich, St. Louis, MO).

### Influenza viruses

Influenza virus A/PR/8/1934 (PR8) (H1N1) was propagated in the allantoic cavity of embryonated chicken eggs at 37°C for 48 hrs. Influenza virus A/Philippines/2/1982/X-79 (H3N2): PR8 (H1N1), a 2:6 recombinant influenza virus containing HA and NA from the influenza A/Philippines/1982 and all the other segments from influenza virus PR8, and the mouse-adapted influenza A/Netherlands/602/2009 (pH1N1) were kindly provided by Drs. F. Krammer and P. Palese (Mount Sinai School of Medicine, New York, NY). All viruses were titrated on MDCK cells in the presence of L-1-tosylamido-2-phenylethyl chloromethyl ketone (TPCK)-trypsin (Sigma-Aldrich), as previously described [26]. Resulting virus stocks were aliquoted and stored at −80°C until use. The LD_50_ of the influenza viruses was calculated in female BALB/c mice by the method of Reed and Muench [27]. All experiments were reviewed and approved by the institutional biosafety program at Weill Medical College of Cornell University.

### Plasmids

The IN-competent packaging vector, pCMVΔR8.2 [6] obtained from Dr. I. Verma (Salk Institute, La Jolla, CA), produces all HIV-1 viral proteins with the exception of the envelope (Env), and including the wild-type IN. The IN-defective packaging vector pCHelp/IN- [28], [29], obtained from Dr. J. Reiser (U.S. Food and Drug Administration, Bethesda, MD), produces all HIV-1 viral proteins with the exception of Env, and contains a point mutation (D116N) in the open reading frame of the IN gene that inactivates the functions characteristic of the IN protein [10]. The envelope-expressing vector, pMD.G [6] obtained from Dr. D. Trono (University of Lausanne, Switzerland), produces VSV-G. The self-inactivating lentiviral transfer plasmids used in this study contain all necessary packaging elements, the central polypurine tract (cPPT), and a CMV promoter that controls the expression of NP (pTY2-CMV-NP), GFP (pTY2-CMV-GFP), or no protein (pTY2-CMV-empty). The pTY2-CMV-GFP and pTY2-CMV-empty vectors have already been described [13], [30]. By further cloning a KpnI/XhoI fragment of DNA containing the coding sequence of the NP protein from influenza virus PR8 in place of GFP, pTY2-CMV-NP was produced. pCAGGS-NP, which was used for this cloning of NP into the transfer vector, was a gift of Dr. A. García-Sastre (Mount Sinai School of Medicine). In order to improve the stability and expression of vector transcripts [31], a woodchuck hepatitis virus posttranscriptional regulatory element (WPRE) was added to the 3′ untranslated region of our gene of interest in the transfer constructs to produce pTY2-CMV-GFP_WPRE_, as previously described [32]. The plasmid, pTY2-CMV-NP_WPRE_, was subsequently obtained by cloning an AgeI/EcoRV fragment of DNA containing the coding sequence of the NP protein in place of the GFP gene in pTY2-CMV-GFP_WPRE_. Primers for the generation of the described plasmid constructs are available upon request. All plasmid constructs were verified by DNA sequencing.

### Construction of lentiviral vectors

**Figure 1:**
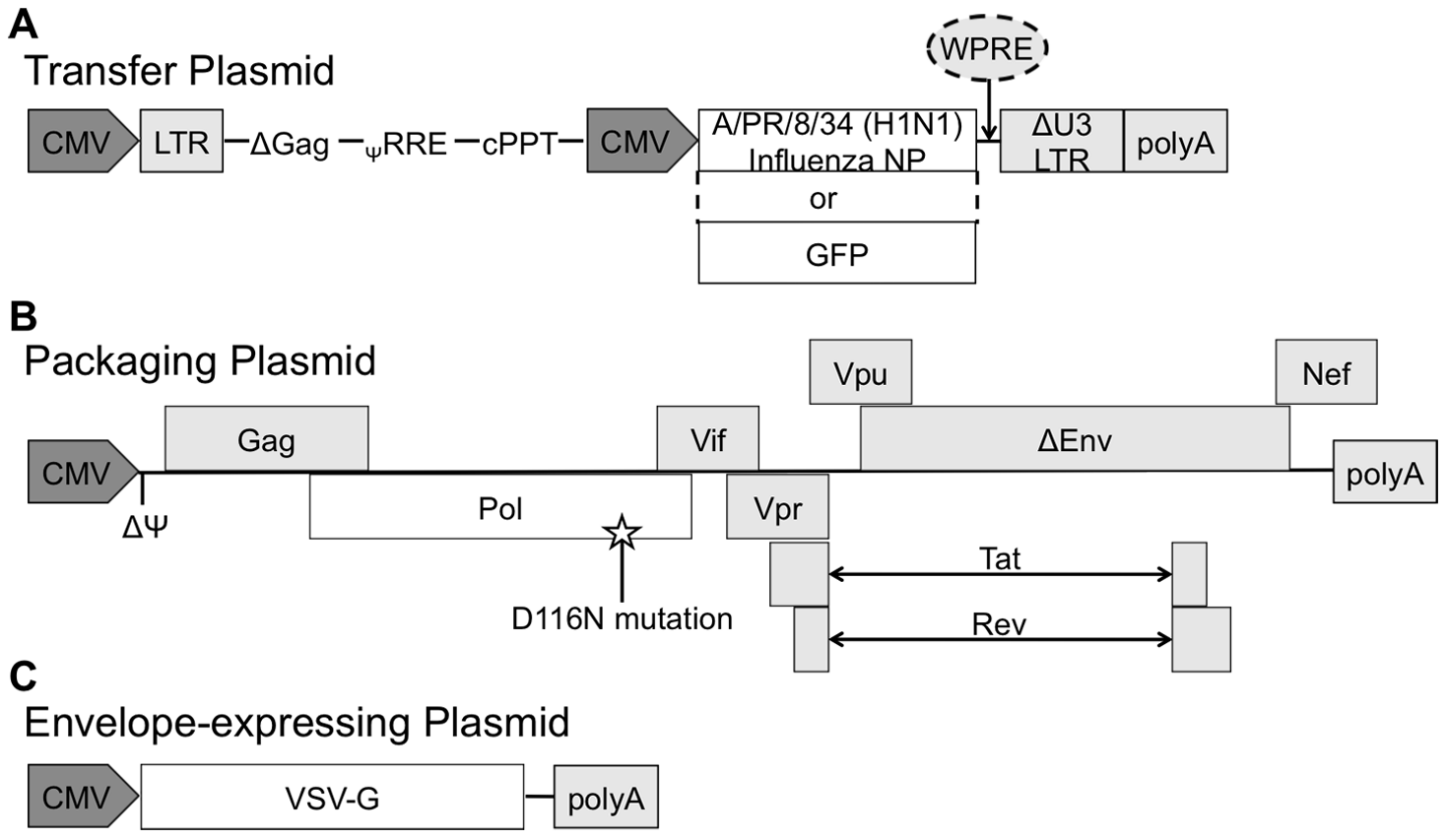
Schematic of lentiviral vectors. (**A**) Lentiviral transfer plasmid that expresses either NP (pTY2-CMV-NP), GFP (pTY2-CMV-GFP) or no exogenous protein (pTY2-CMV-empty). The arrow indicates where a woodchuck hepatitis virus posttranscriptional regulatory element (WPRE) is added for the production of the pTY2-CMV-NP_WPRE_ and pTY2-CMV-GFP_WPRE_ constructs. (**B**) Lentiviral packaging plasmid, pCHelp/IN- and pCMVΔR8.2, which are differentiated by the presence of the mutation D116N that inactivates the function of the integrase protein in pCHelp/IN- (indicated by a star). (**C**) Envelope-expressing plasmid, pMD.G, which produces the vesicular stomatitis G protein (VSV-G). Abbreviations: cytomegalovirus promoter (CMV), long terminal repeat (LTR), deleted gag gene (ΔGag), packaging signal (Ψ), rev responsive element (RRE), central polypurine tract (cPPT), nucleoprotein (NP), green fluorescent protein (GFP), deleted unique 3′ region (ΔU3), bovine growth hormone polyadenylation signal (polyA), deleted packaging signal (ΔΨ), nonfunctional envelope (ΔEnv).

IN-defective (ID) and IN-competent (IC) lentiviral vectors (LV) were generated by co-transfection of 3 plasmids (Figure 1), as previously described [28]: (1) the pCHelp/IN- or pCMVΔR8.2 packaging vector, (2) the pTY2-CMV-NP (+/− WPRE), pTY2-CMV-GFP (+/− WPRE), or the pTY2-CMV-empty transfer vector, and (3) the pMD.G envelope vector. For the transfection, 293T cells were plated on 100 mm TC-treated culture dishes (Corning, Tewksbury, MA) coated with 0.8% gelatin (Sigma-Aldrich) and 0.002% poly L-lysine (Sigma-Aldrich) in PBS, and incubated overnight. Cells were transfected with the 3 plasmids described above using the Profection Mammalian Transfection System (Promega Corporation, Madison, WI), and media was changed after 10 hrs. At 48 and 72 hrs post-transfection, culture supernatants were collected, cleared of cellular debris by centrifugation, filtered through a 0.45 µm pore-sized PVDF filter (EMD Millipore, Billerica, MA), and concentrated on a 20% sucrose gradient (Sigma-Aldrich) by ultracentrifugation at 27,000 rpm (∼131,100×g) for 2 hrs at 4°C using an AH-629 swinging bucket rotor (Thermo Scientific) in a Sorvall WX Ultra 80 ultracentrifuge (Thermo Scientific). Pellets were resuspended in PBS and stored at −80°C until use. Viral titers were determined by flow cytometric measurement of NP or GFP protein expression in 293T cells transduced with concentrated LV and expressed as transducing units per mL (TU/mL), calculated as previously described [33].

### Flow Cytometry

293T cells were plated in 6-well microplates and incubated overnight. Cells were then counted and transduced with LV in DMEM with 5% FBS overnight. Following transduction with LV, media was changed and cells were further incubated 1 or 2 days until time of assay. Cells transduced with GFP-expressing LV were fixed with 4% paraformaldehyde and assayed directly, whereas cells transduced with NP-expressing LV were fixed and permeabilized using the BD Cytofix/Cytoperm kit (BD Biosciences, San Diego, CA), then stained with a FITC-conjugated anti-influenza A NP monoclonal antibody (MA1-7322, Thermo Scientific). GFP or FITC fluorescence in transduced cells was measured using a Becton-Dickinson FACScan (BD Biosciences), and data were analyzed using FlowJo v9.4.11.

### Western blot

LV-transduced or influenza-infected 293T cells were lysed in RIPA buffer (1% Sodium deoxycholate, 1% Triton X-100, 0.2% SDS, 150 mM NaCl and 50 mM Tris-HCl, pH 7.5 in ddH_2_O) containing the Complete mini protease inhibitor cocktail (Roche Diagnostics, Florham Park, NJ), and protein was quantitated using the Pierce BCA Protein Assay kit (Thermo Scientific). Equivalent amounts of protein from each sample were run on a Mini-PROTEAN TGX, 4–15%, 10-well SDS-PAGE gel (Bio-Rad Laboratories, Inc., Hercules, CA) and probed by Western blot using a rabbit anti-influenza A NP polyclonal antibody (Thermo Scientific), a rabbit anti-GAPDH polyclonal antibody (FL-335, Santa Cruz Biotechnology, Inc., Dallas, TX) and an HRP-conjugated donkey anti-rabbit IgG (H+L) secondary antibody (Abcam, Cambridge, England).

### Mouse Immunizations and Challenge Experiments

Female BALB/c mice, 6–8 weeks old, were purchased from Charles River (Wilmington, MA) or Jackson Laboratories (Bar Harbor, ME). Mice anesthetized with inhalational isoflurane were immunized with LV expressing NP or GFP via either the i.m. (3−10×10^5^ TU/mouse) or i.n. (1−3×10^5^ TU/mouse) route of administration. LV were administered in 100 µL total volume when administered i.m., and 50 µL when administered i.n. reflecting, in the latter case, the total respiratory tract immunization [34].

Mice were immunized and boosted in accordance with various protocols, and challenged 4–6 weeks following the final immunization with lethal doses of influenza virus in 50 µL PBS administered i.n. while under anesthesia with isoflurane. Body weights were measured daily. Mice were euthanized when they were moribund (weight loss equal to ≥20% of their initial body weight).

### IFN-γ ELISPOT

Spleens were harvested from mice at various time points after immunization with IDLV, and the number of IFN-γ-secreting T cells after restimulation with H-2k^d^-restricted CD8+ T cell epitopes were evaluated using an IFN-γ ELISPOT assay (Mabtech, Sweden). Briefly, spleens were passed through 40 µm nylon cell strainers (BD Biosciences), treated with ammonium chloride potassium (ACK lysis buffer; Lonza) and resuspended in Complete RPMI media to obtain single-cell suspensions. Splenocytes were restimulated in triplicate in an IFN-γ ELISPOT plate using immunodominant H-2k^d^-restricted 9-mer peptides: TYQRTRALV (amino acids 147–155) for NP [21], [35] or HYLSTQSAL (amino acids 200–208) for GFP [36]. The NP and GFP peptides were synthesized by the Proteomics Resource Center of Rockefeller University, New York, NY. Concanavalin A (Sigma-Aldrich) is a polyclonal stimulator of T cell activation, and was therefore used as a positive control to verify assay functionality. The plate was developed according to manufacturer's protocol, and spot-forming cells were counted with an automated ELISPOT reader (AID ELR02 coupled with AID ELISPOT software, version 5.0). Values obtained from cells restimulated with an unrelated peptide were subtracted from values obtained from cells restimulated with a specific peptide, and the final numbers were expressed as spots per million cells.

### NP-specific serum IgG ELISA

Serum was collected from each mouse by submandibular bleeding at various time points pre- and post-immunization with LV and frozen at −20°C until time of assay. The NP-specific serum IgG ELISA was performed as previously described [37]. Briefly, Nunc flat-bottom 96-well polystyrene microplates (Thermo Scientific) were coated with 1 µg/mL of recombinant influenza A NP (Imgenex Corporation, San Diego, CA) in PBS and incubated overnight at 4°C. Plates were blocked at room temp. for 2 hrs using PBS with 0.05% sodium azide (Teknova, Hollister, CA) supplemented with 10% FBS. Following 3 washes with PBS supplemented with 0.5% Tween-20, mouse sera were serially diluted in diluent buffer (0.05% sodium azide, 0.25% BSA and 0.05% Tween-20 in PBS), transferred into the NP-coated plate (100 µL), and incubated at room temp. for 2 hrs. Plates were washed again, and alkaline phosphatase-conjugated goat anti-mouse IgG secondary antibodies (Southern Biotechnology, Birmingham, AL) diluted in diluent buffer were added. After 1 hr of incubation at room temp., plates were washed, and 100 µL of AKP substrate (1 mg/mL 4-Nitrophenyl phosphate disodium salt hexahydrate (Sigma-Aldrich), 50 mM NaHCO3, 1 mM MgCl2, pH 9.8 in ddH_2_O) was added to each well. Plates were incubated 60 min., and OD was measured with a PR 3100 TSC microplate reader (Bio-Rad Laboratories, Inc.) at 405 nm. The starting dilution was 1:25 for all serum samples, with serial twofold dilutions thereafter. Sera with no activity at 1:25 were assigned titers of 1:12.5. Endpoint titers were defined as the reciprocal of the highest dilution that gave an OD reading three standard deviations above the mean of the pre-immunization sera.

### Statistical methods

Antibody titer data were log-transformed prior to statistical analysis. Mean/median percent initial bodyweight was compared between groups, at each time point, using the Kruskal-Wallis test or the ANOVA test, as appropriate. Similar comparative analyses between groups were performed for ELISPOT measurements and antibody titers. Pairwise group comparisons (following ANOVA tests) were not adjusted for multiple comparisons due to the exploratory (i.e. hypothesis-generating) nature of this pilot study. Kruskal-Wallis tests and ANOVA tests yielded similar results, so all pairwise (i.e. post-hoc) analyses were based on ANOVA analyses for readability. All p-values are two-sided with statistical significance evaluated at the 0.05 alpha level. All analyses were performed in SPSS Version 21.0 (SPSS Inc., Chicago, IL).

### Ethics Statement

All animal procedures were performed in strict accordance with Institutional Animal Care and Use Committee (IACUC) guidelines, and have been approved by the IACUC of the Joan & Sanford I. Weill Medical College of Cornell University (Protocol Number: 2009-045). All procedures were performed under inhalational isoflurane anesthesia, and every effort was made to minimize suffering.

## Results

### Generation and analysis of lentiviral vectors expressing NP

To generate LV expressing NP, we first constructed the transfer vector, pTY2-CMV-NP, by inserting the PR8 NP gene into the lentiviral transfer vector, pTY2-CMV. Correct orientation of the NP gene was confirmed by full NP sequencing (data not shown). The pTY2-CMV-NP plasmid (Figure 1A) was then co-transfected with either the IN-defective (pCHelp/IN-) or the IN-competent (pCMVΔR8.2) packaging plasmid (Figure 1B), and the envelope-expressing plasmid, pMD.G (Figure 1C), to make either IDLV expressing NP (IDLV-NP) or ICLV expressing NP (ICLV-NP), respectively. We also generated LV expressing GFP (IDLV-GFP or ICLV-GFP) or lacking a gene insert (IDLV-empty or ICLV-empty) to be used as controls.

**Figure 2:**
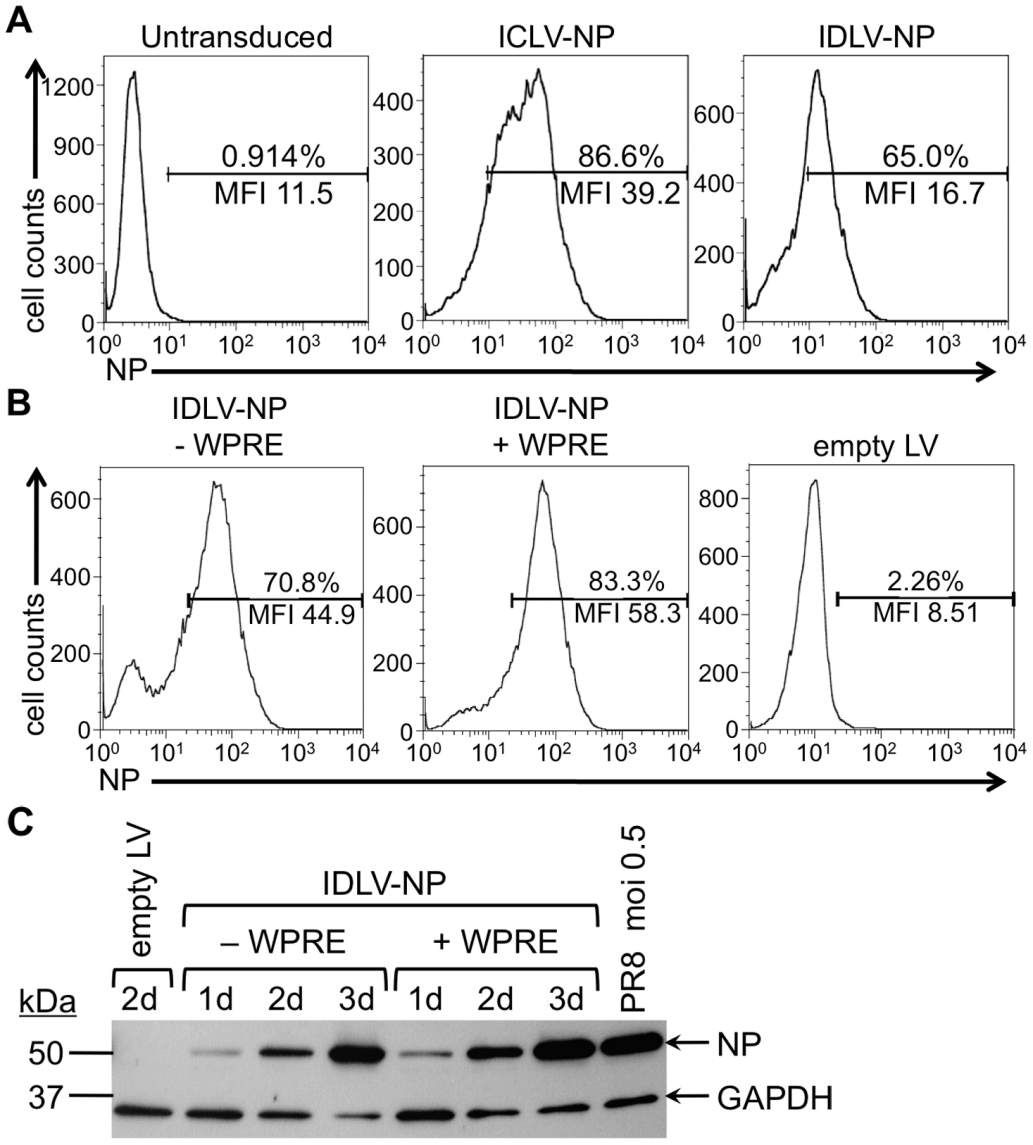
IDLV express NP protein *in vitro*. (**A**) 293T cells transduced with IDLV-NP or ICLV-NP with a multiplicity of infection (MOI) of 3 were fixed and stained at day 2 post-transduction with a FITC-conjugated antibody against influenza A virus NP, then measured by flow cytometry. Untransduced cells were used as a negative control. The percentage of FITC-positive cells and mean fluorescence intensity (MFI) in each sample is indicated. (**B**) 293T cells were transduced for 1 day with IDLV-NP (+ or − WPRE) (MOI = 3), fixed and stained with a FITC-conjugated antibody against influenza A virus NP, and then measured by flow cytometry. The percentage of FITC-positive cells and MFI in each sample is indicated. ICLV-empty was used as a negative control (empty LV). (**C**) Lysates from cells transduced as described in panel B were collected on day 1, 2 or 3, and probed for influenza virus NP (∼56 kDa) by western blot. Equivalent amounts of protein (9 µg) from each sample were loaded. Lysate from cells infected with PR8 influenza virus (MOI = 0.5) was used as a positive control for protein expression.

We then assessed the ability of IDLV to transduce cells and express proteins *in vitro*, in comparison to their integrating counterparts. 293T cells were transduced with IDLV and ICLV expressing either GFP or influenza NP, and protein expression was evaluated visually by fluorescence microscopy for GFP (not shown), and quantitatively by flow cytometry for NP (Figure 2A). We found that, in both cases, cells were efficiently transduced (65.0% or 86.6% of cells positive for NP expression 2 days after transduction with IDLV-NP or ICLV-NP, respectively) and proteins were expressed by IDLV, albeit, as expected, at lower levels than those expressed from ICLV (MFI of 16.7 versus 39.2 for IDLV-NP or ICLV-NP, respectively) (Figure 2A).

Since adding the *cis*-acting woodchuck hepatitis virus post-transcriptional regulatory element (WPRE) to the 3′ untranslated region of genes expressed from lentiviral vectors can enhance the expression of the transcript by several fold [38], we tested whether the presence of WPRE in pTY2-CMV-NP would increase protein expression from IDLV-NP. For this purpose, we cloned WPRE into our transfer vector to obtain pTY2-CMV-NP_WPRE_ (Figure 1A) and generated IDLV-NP (+ WPRE). We then transduced 293T cells with either IDLV-NP (+ WPRE) or IDLV-NP (- WPRE), and compared NP protein expression by flow cytometry (Figure 2B) and by western blot at various times post-transduction (Figure 2C). NP expression from vectors with WPRE was higher than expression from vectors without WPRE, supporting our decision to perform all subsequent experiments using IDLV (+ WPRE), designated hereafter as IDLV-NP or IDLV-GFP.

### Proteins produced by IDLV induce dose-dependent and persistent cell-mediated immune responses *in vivo*

**Figure 3:**
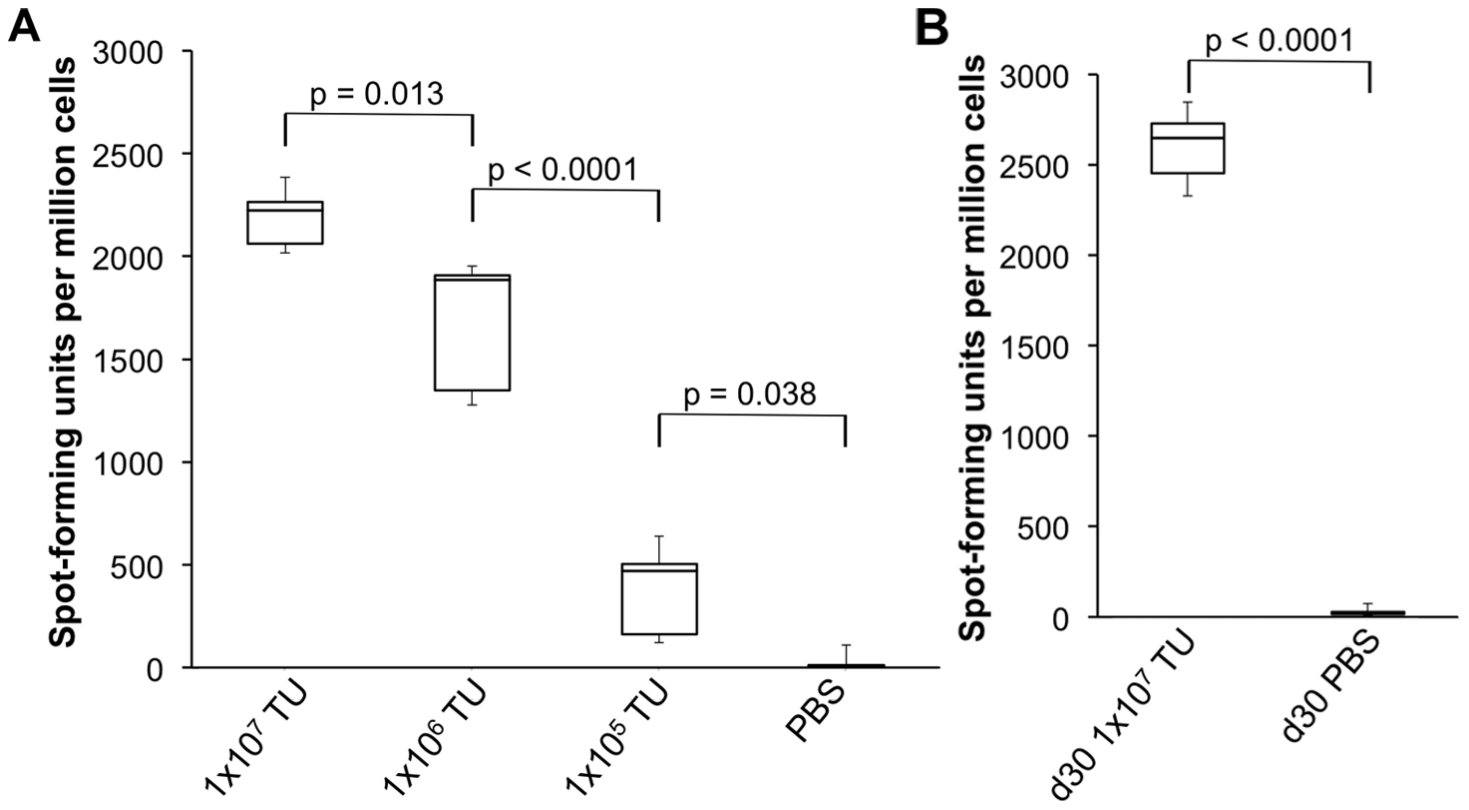
IDLV generate dose-responsive and antigen-specific T cell responses in mice. Groups of BALB/c mice (n = 3) were immunized i.m. with varying doses (TU/mouse) of IDLV-GFP, as indicated. Splenocytes were assayed on day 10 (**A**) or day 30 (**B**) post-immunization by ELISPOT for IFN-γ responses to an H-2k^d^-restricted 9-mer GFP peptide, or to an unrelated peptide (not shown). Mice injected with PBS served as a negative control. The Kruskal-Wallis test was used to compare GFP levels between the dosing groups (overall p-value of 0.006). P-values from pairwise dose group comparisons based on the ANOVA test are shown.

To assess whether proteins expressed from IDLV can generate an immune response *in vivo*, and to identify which dose(s) of IDLV elicit strong responses, we measured antigen-specific T cell responses after administering varying doses of IDLV-GFP to BALB/c mice by the i.m. route. At day 10 post-immunization, mice were euthanized, and splenocytes were harvested and restimulated with a 9-mer peptide corresponding to an H2-K^d^-restricted CD8+ T cell epitope of GFP (GFP_200–208_) [36], or to an unrelated H2-K^d^-restricted 9-mer CD8+ T cell epitope (not shown). Interferon-γ (IFN-γ) production from mouse splenocytes was measured by ELISPOT. Mice immunized with higher doses of IDLV-GFP showed significantly higher IFN-γ responses to the GFP peptide (Figure 3A), while T cell responses to the unrelated peptide were negligible, and similar to the blank control (not shown). Control mice that had been injected with PBS showed no measurable response. This result suggests that proteins produced by IDLV are specifically recognized by the immune system in a dose-dependent manner. In addition, T cell responses were still elevated one month after immunization (Figure 3B), suggesting that a single immunization with IDLV is capable of generating a long-lasting cell-mediated response *in vivo*. To explore whether IDLV-GFP could also induce an immune response against the expressed antigen when administered by the i.n. route, and to compare this response to that obtained by i.m. administration, we administered 10^7^ TU of IDLV-GFP to BALB/c mice by the i.n. route in the same experiment. Mice were euthanized 10 days after immunization, as previously described, and splenocytes were harvested and restimulated with the GFP_200–208_ peptide. We found that the GFP-specific T cell responses in the spleen of mice that received IDLV-GFP by the i.n. route were significantly lower (p<0.001) than those measured after i.m. administration (Figure S1).

**Figure 4:**
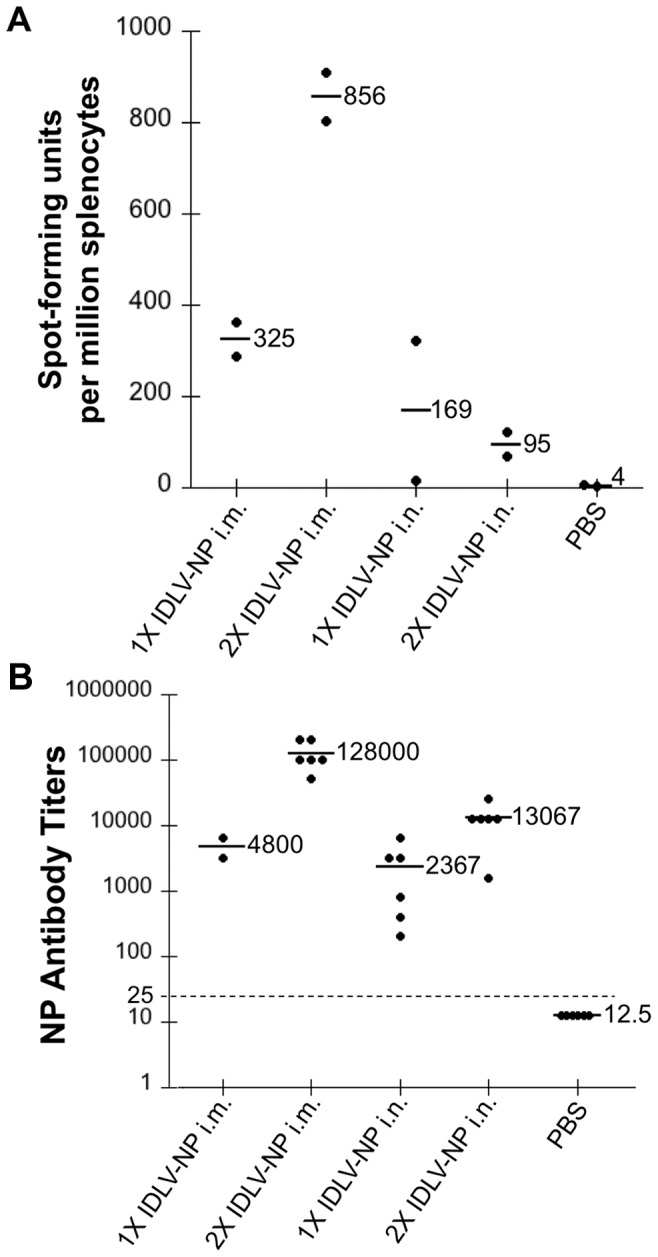
Immunization with IDLV-NP induces NP-specific cell-mediated and humoral immune responses. Separate groups of mice were inoculated either once (1X) or twice (2X), 4 weeks apart, with IDLV-NP via either the i.m. or i.n. route of administration. Mice that were injected i.m. with PBS served as a negative control. (**A**) Splenic T cell responses were evaluated 4 weeks following the final immunization by IFN-γ ELISPOT after restimulation with the immunodominant H-2k^d^-restricted 9-mer NP peptide, or with an unrelated H-2k^d^-restricted 9-mer peptide (not shown). (**B**) Serum was collected from each mouse prior to the first immunization and 4 weeks after the last immunization. NP-specific IgG in serially-diluted serum samples was measured by indirect ELISA assay and is expressed as endpoint titer. The lower limit of detection is indicated by the dotted line. For all panels, each dot represents one mouse, and bold lines indicate the mean.

We next investigated the ability of IDLV to induce a specific immune response directed against the NP protein. For this purpose, groups of BALB/c mice were immunized, either once or twice, 4 weeks apart, with IDLV-NP via either the i.n. or i.m. route of administration. Mice injected twice with PBS were used as a negative control. T cell responses to the immunodominant, H-2k^d^-restricted 9-mer NP peptide (NP_147–155_) were measured in mouse splenocytes (Figure 4A) by IFN-γ ELISPOT, 4 weeks after the final immunization. As a specificity control, IFN-γ responses to restimulation with an unrelated H-2k^d^-restricted peptide (GFP_200–208_) were also measured.

We found that mice receiving IDLV-NP by either route were able to mount an NP-specific T cell response at the level of the spleen. Similarly to IDLV-GFP, mice immunized by the i.m route had higher T cell responses than mice inoculated by the i.n. route. Splenocytes did not respond to the unrelated peptide or to the blank control (not shown).

### IDLV-NP elicit NP-specific antibody responses when administered by i.n. and i.m. routes *in vivo*

We next investigated the ability of IDLV to initiate an antibody response directed against NP after either i.n. or i.m. administration. Mice immunized as described above were bled prior to immunization and 4 weeks after immunization, and ELISA was used to test sera for the presence of total NP-specific IgG. As observed with T cell responses, IDLV-NP administration by either route was able to induce a robust NP-specific antibody response, and that response was more elevated in mice immunized by the i.m. route and in mice that received two doses of IDLV-NP, compared to mice that only received a single administration (Figure 4B). As expected, PBS did not induce NP-specific IgG.

### NP-specific antibody responses are boosted by IDLV-NP readministration

**Figure 5:**
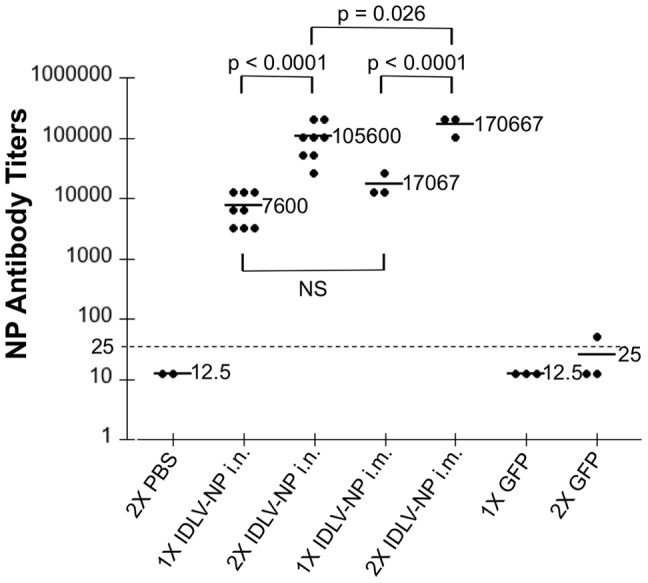
Immunization with IDLV-NP induces NP-specific IgG responses that are increased upon boosting. Mice were inoculated 2 times i.n. or i.m. with IDLV-NP, 4 weeks apart. Mice that received either PBS or IDLV-GFP 1 or 2 times i.n. served as a negative control. Serum was collected from each mouse prior to the first immunization, prior to the second immunization (1X), and 4 weeks after the second immunization (2X). NP-specific IgG in serially-diluted serum samples was measured by indirect ELISA assay and is expressed as endpoint titers, defined using the pre-immunization sera as a baseline. Each dot in the panel represents one mouse, and bold lines indicate the mean. The lower limit of detection is indicated by the dotted line. The Kruskal-Wallis test was used to compare antibody titers between the groups (overall p-value of <0.0001). P-values from pairwise group comparisons based on the ANOVA test are shown.

A limitation of some viral vectors is the inability to readminister them due to host anti-vector immune responses that are preexisting or arise following administration. Since in the previous experiment we showed that levels of NP-specific IgG were higher in mice that received 2 doses of the vector than in mice that had received only one dose, we next tested whether NP-specific antibodies could be boosted by readministration of IDLV-NP to the same mouse. BALB/c mice were immunized either twice i.n. or twice i.m. with IDLV-NP, and were bled prior to the first immunization, prior to the second immunization and 4 weeks after immunization. ELISA was used to measure levels of total NP-specific IgG in the sera. Mice inoculated twice i.n. with IDLV-GFP served as specificity control, and mice that received PBS two times i.n. served as a negative control. As expected, mice immunized with IDLV-NP by either route of administration mounted a robust IgG antibody response against NP (Figure 5). We further found that, also regardless of the route, the NP-specific IgG response was significantly increased after readministration of IDLV-NP (Figure 5). By contrast, IDLV-GFP and PBS did not induce any NP-specific IgG (Figure 5). This result suggests that IDLV-NP can be readministered to induce a significant increase in the antigen-specific humoral immune response.

### Administration of IDLV-NP protects mice from lethal homologous influenza virus challenge

**Figure 6:**
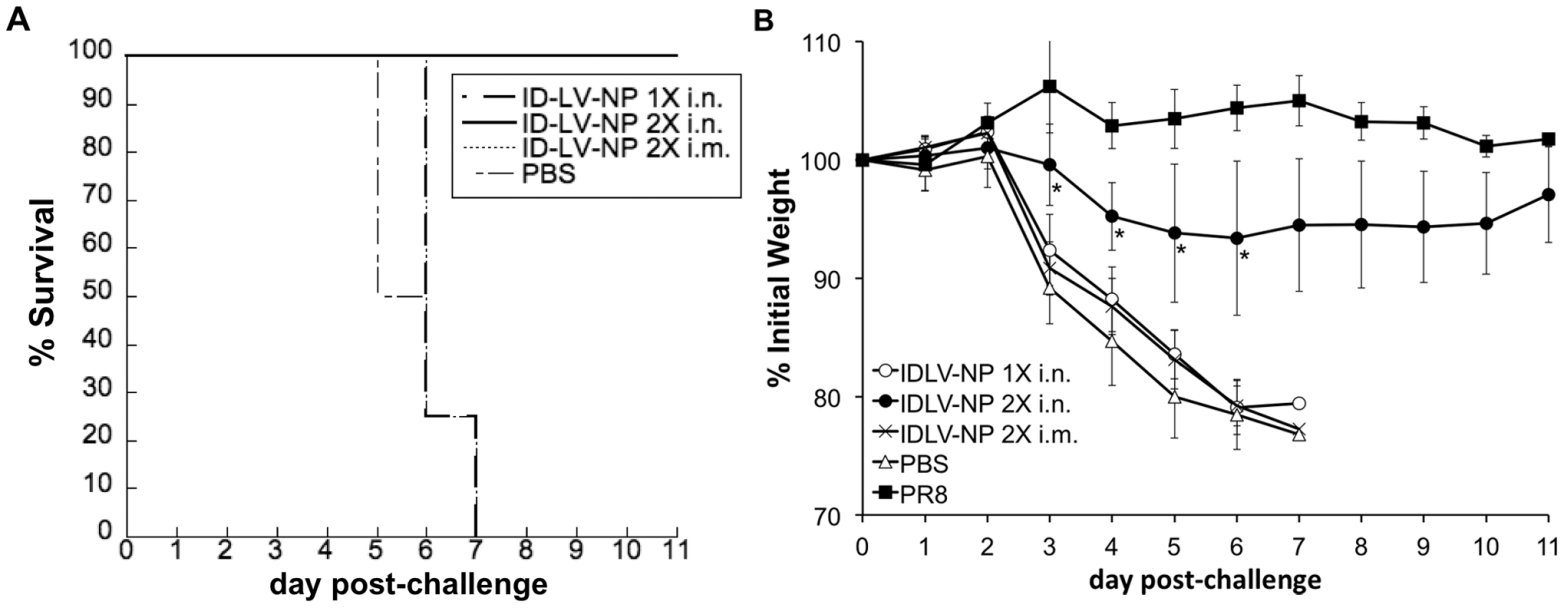
Immunization with IDLV protects mice from homologous influenza challenge. Groups of mice (n = 4) were immunized 1 or 2 times, 4 weeks apart, with IDLV-NP via either the i.m. or i.n. route of administration. Mice that were injected i.m. with PBS served as a negative control, and mice that were infected i.n. with a sublethal dose of PR8 influenza virus served as a positive control for protection. Survival (**A**) and weight loss (**B**) were monitored for 11 days following i.n. challenge with a lethal dose (10 LD_50_) of PR8 influenza virus 4 weeks after the final inoculation. Weight loss is presented as the average percentage of initial weight at the time of challenge. *indicates days when IDLV-NP 2X i.n. lost significantly less weight than any of the other immunization regimens (p<0.001, calculated using pairwise group comparisons, at each time point, based on ANOVA). Overall p-value for group comparisons was based on the Kruskal-Wallis test: p = 0.001 for day 3, p = 0.002 for day 4, p = 0.005 for day 5, p = 0.008 for day 6.

In the previous experiments, we determined that IDLV-NP, when administered by the i.n. route, can induce NP-specific T cell and IgG antibody responses. Therefore, we wanted to determine whether i.n. administration of IDLV-NP was also able to protect mice from lethal challenge with a homologous influenza A virus, and we wanted to further compare the effectiveness of the i.n. and i.m. routes of administration. To answer these questions, BALB/c mice were immunized by administering IDLV-NP either i.n (once or twice, one month apart) or i.m. (twice, one month apart). One month after the last IDLV-NP administration, mice were challenged i.n. with a lethal dose (10 LD_50_) of PR8 influenza virus, the same strain as that used to clone NP into IDLV-NP. Animals were monitored daily, and were euthanized if they lost 20% of their initial body weight or appeared to be moribund. Mice that received 2 i.n. doses of IDLV-NP were fully protected (Figure 6A) and showed no or minimal weight loss (Figure 6B), while the other vaccine combinations were unable to effectively protect the mice from disease and death. As expected, all animals that had been immunized with a sublethal dose of PR8 influenza virus as a positive control for protection survived the challenge (not shown), while all mice that were mock-immunized with PBS succumbed to influenza infection.

**Figure 7:**
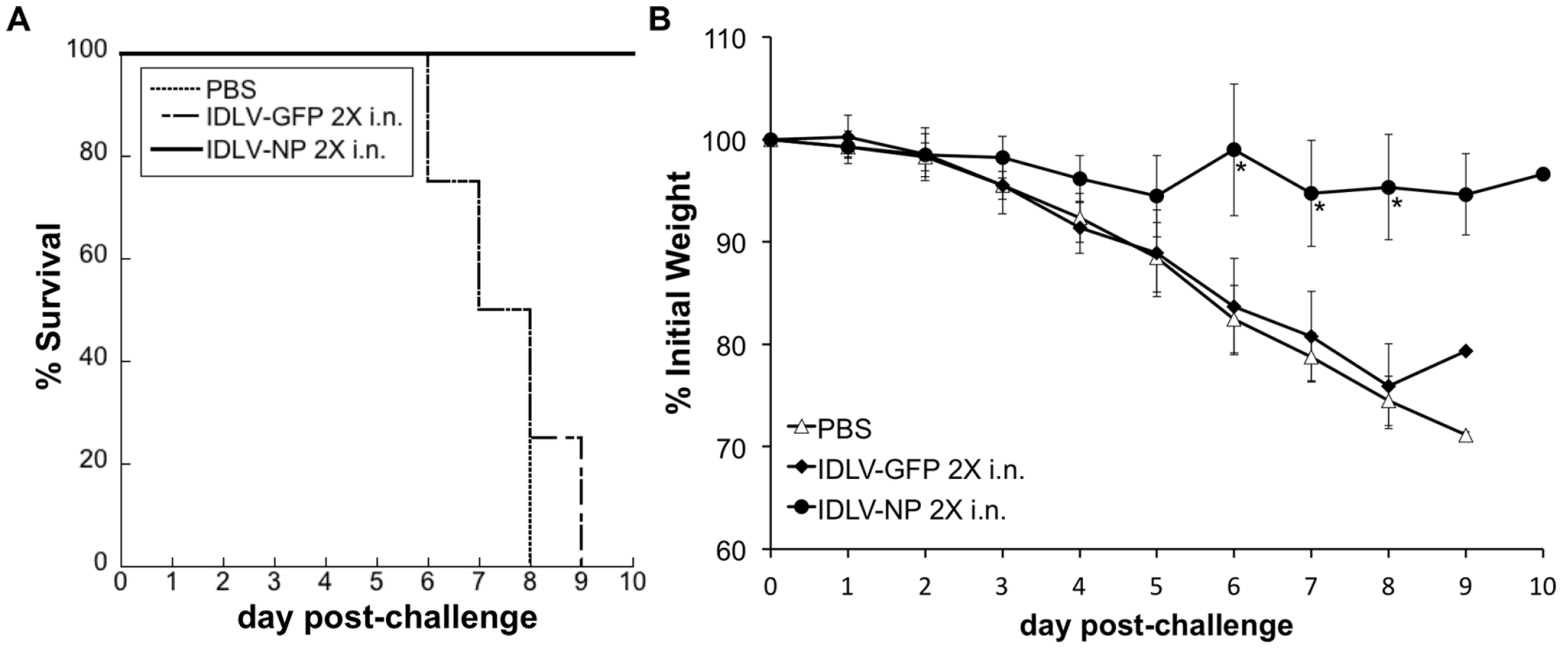
Immunization with IDLV protects mice from challenge with influenza virus in an antigen-specific manner. Mice were inoculated 2 times i.n., 4 weeks apart, with IDLV-NP (n = 5) or IDLV-GFP (n = 4). Mice that received PBS 2 times i.n. (n = 4) served as a negative control for protection. Four weeks after the final administration, mice were challenged with a lethal dose (5 LD50) of the mouse-adapted A/Philippines/1982:PR8 influenza virus. Survival (**A**) and weight loss (**B**) were monitored for 10 days following challenge. Weight loss is presented as the average percentage of initial weight at the time of challenge. *indicates days when IDLV-NP 2X i.n. lost significantly less weight than any of the other immunization regimens (p<0.005, calculated using pairwise group comparisons, at each time point, based on the ANOVA test). Overall p-value for group comparisons was based on the Kruskal-Wallis test: p = 0.029 for day 6, p = 0.021 for day 7, p = 0.026 for day 8.

We next tested whether the protection induced by IDLV-NP was specific, and not due to non-specific anti-vector immunity. Since, in the previous protection experiment, the i.n. route was superior to the i.m. route regarding induction of protective immunity, we focused on this route of administration. Groups of BALB/c mice were immunized with either IDLV-NP, or IDLV-GFP as a specificity control to rule out innate immune protection due to the vector. Mice that received PBS served as negative control for protection. All groups received 2 i.n. doses of IDLV or PBS, 4 weeks apart. Four weeks after the final immunization, mice were challenged with a lethal dose (5 LD_50_) of A/Philippines/2/1982/X-79 (H3N2): PR8 (H1N1) influenza virus, a 2:6 reassortant with the same NP gene as PR8 influenza virus. Again, all mice immunized with IDLV-NP were protected from death and showed no significant weight loss (Figure 7A,B). Conversely, mice that received PBS or IDLV-GFP exhibited rapid weight loss and succumbed to disease by day 9 post-infection. In separate experiments, we immunized mice using IDLV-empty to control for antigen specificity, and we had similar results, with all mice treated with IDLV-empty succumbing to lethal challenge (not shown). These data suggest that the protection conferred by IDLV-NP is specific, and not secondary to vector-related immunity.

### Immunization with IDLV-NP confers cross-protection from lethal heterosubtypic challenge with H1N1 pandemic influenza virus

**Figure 8:**
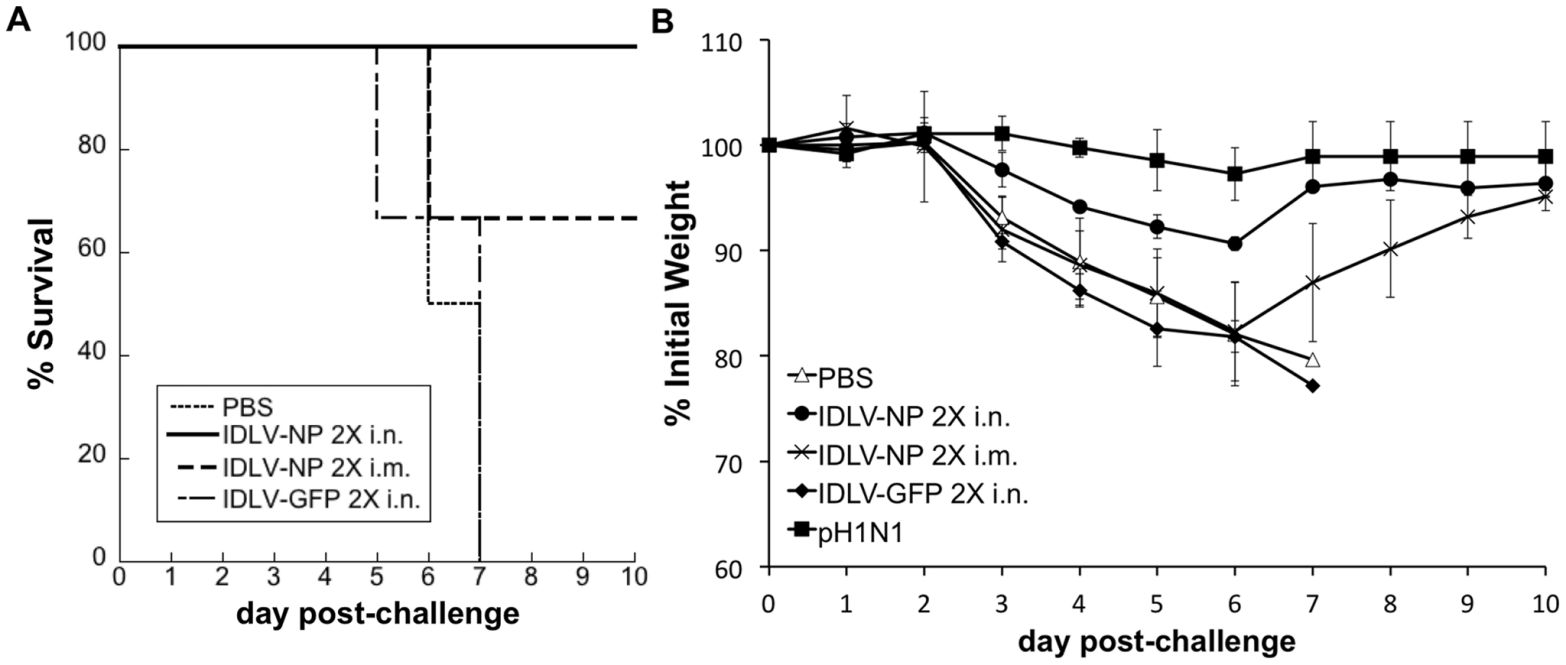
Immunization with IDLV-NP protects mice from challenge with a heterosubtypic influenza virus. Groups of mice (n = 3) were inoculated with IDLV-NP 2 times i.n. or i.m., 4 weeks apart. Mice (n = 3) that received PBS 2 times i.n. served as a negative control, mice (n = 3) inoculated with 2 i.n. doses of IDLV-GFP, 4 weeks apart, served as a specificity control, and mice (n = 3) that were infected with a sublethal dose of the mouse-adapted A/Netherlands/602/2009 (pH1N1) influenza virus served as a positive control for protection. Six weeks after the final administration, mice were challenged with a lethal dose (>10 LD_50_) of pH1N1 influenza virus and monitored for survival (**A**) and weight loss (**B**). P-values calculated using pairwise group comparisons, at each time point, were based on the ANOVA test. For weight comparisons, Day 3: p<0.05 IDLV-NP 2X i.n. versus PBS; p<0.01 IDLV-NP 2X i.n. versus IDLV-GFP 2X i.n. or IDLV-NP 2X i.m.; Day 4: p<0.05 IDLV-NP 2X i.n. versus PBS and IDLV-NP 2X i.m.; p<0.005 IDLV-NP 2X i.n. versus IDLV-GFP 2X i.n.; Day 5, p<0.05 IDLV-NP 2X i.n. versus IDLV-NP 2X i.m.; p = 0.005 IDLV-NP 2X i.n. versus IDLV-GFP 2X i.n. Overall p-value for group comparisons based on the Kruskal-Wallis test: p = 0.024 for day 3, p = 0.029 for day 4, p = 0.027 for day 5.

To evaluate whether IDLV-NP could provide cross-protection against challenge with a heterosubtypic influenza virus strain with pandemic potential, BALB/c mice were immunized with 2 doses of IDLV-NP, and then challenged 4 weeks after last administration with 10 LD50 (2×10^4^ pfu) of a mouse-adapted A/Netherlands/602/2009 (pH1N1) influenza virus, and monitored for weight loss and survival. Mice administered 2 i.n. doses of PBS were used as a negative control and mice immunized with 2 sublethal i.n. doses of pH1N1 were used as a survival control. All mice immunized 2 times i.n. with IDLV-NP showed minimal weight loss during the time of the experiment and survived challenge (Fig S2A,B). All mice that were immunized with a sublethal dose of pH1N1 as a positive control survived and lost no significant weight, as expected (Figure S1B). Mice that were mock immunized with PBS rapidly lost weight and died, except for one of them who, although close to the cut-off, regained weight and survived challenge. We therefore repeated the experiment using 2.5×10^4^ pfu of pH1N1 influenza virus as lethal challenge. In this experiment, BALB/c mice were immunized with 2 doses of IDLV-NP, 4 weeks apart via either the i.m. or i.n. route of administration. Mice immunized with 2 i.n. doses of IDLV-GFP were used as a specificity control, and mice administered 2 i.n. doses of PBS were used as a negative control. Mice were challenged 6 weeks after the second vaccination with a lethal dose of the pH1N1 influenza virus, and monitored for weight loss and survival. As expected, mice immunized with PBS or GFP lost significant body weight, and died by day 7 after challenge (Figure 8A,B). In contrast, all mice immunized 2 times i.n. with IDLV-NP showed minimal weight loss during the time of the experiment and survived challenge (Figure 8A,B). Some of the mice that were immunized i.m. with IDLV-NP survived the influenza challenge as well; however, these animals experienced significant weight loss (Figure 8A,B). All mice that were immunized with a sublethal dose of pH1N1 as a positive control survived (not shown) and lost no significant weight, as expected (Figure 8B). These data suggest that administration of IDLV-NP protects from heterosubtypic challenge with pandemic H1N1 influenza virus.

## Discussion

In this study, we assessed the feasibility of administering IDLV by the i.n. route to induce an antigen-specific immune response and protect against influenza virus. IDLV is an attractive platform for vaccine delivery since they, like ICLV, have the potential to robustly activate both the cell-mediated and humoral arms of the immune system [4], [16], [17], [28], [39], but they have the safety advantage of doing so without integrating into the cell genome [11]. We constructed IDLV expressing influenza NP, which is the major target for host T cell responses [21], because these NP-specific responses have been shown to be protective against homologous and heterosubtypic influenza virus challenge in mice [22], [40], [41]. Antibodies against NP may also help to promote resistance to influenza virus infection [25], [42].

Our results clearly indicate that immunization with IDLV-NP strongly induces both cell-mediated and humoral NP-specific immune responses in mice. Furthermore, a repeated dose of IDLV-NP enhance NP-specific immune responses, suggesting either that IDLV-NP did not elicit anti-vector immunity, or that anti-vector immunity did not interfere with the ability of the mice to mount antigen-specific immune responses following vector readministration in this system. This result is consistent with the known lack of pre-existing immunity and negligible host immune response against LV [8]. This is also the first study to demonstrate that IDLV, when administered via the i.n. route, can protect against a viral infection. In our study, the ability of IDLV-NP to confer protective immunity against homologous or heterosubtypic influenza virus challenge was strongly dependent on the route of administration. In fact, full protection was only achieved with 2 doses of IDLV-NP administered by the i.n. route, but not by the i.m. route. Our finding is in agreement with other reports of viral vector-based vaccines expressing NP, or other conserved influenza proteins, that have shown to be more effective when administered by the i.n. route, compared to other routes [43], [44], [45].

The exact correlates of protection associated with the i.n. route of administration have not yet been clearly identified for any of the vectors studied. We also did not observe a clear correlation between increases in NP-specific T cell or serum IgG antibody responses and protection against influenza. Splenic T cell responses were generally more elevated in mice that received IDLV by the i.m. route than in those inoculated by the i.n. route, but these mice were either not protected or were only partially protected from challenge. This was consistent with other reports suggesting that splenic T cell responses alone are not effective at controlling infection with a mucosal threat [46], [47]. Similarly, although serum anti-NP IgG antibody responses were elevated in mice after a single i.n. or i.m. administration, no apparent correlation was evident between these antibody responses and protection against influenza.

Studies using a recombinant adenovirus vector expressing influenza NP and M2 suggested that NP-specific T cells generated in the lung following i.n. inoculation are important mediators of protection [43], [48]. Both i.n. and i.m. immunization with rAdV expressing NP and M2 resulted in an increased number of NP-specific T cells in the lung compared to unimmunized mice [48]. In mice that received rAdV i.n., however, lung cellularity was also increased several fold compared to those who received rAdV i.m, so when the data were recalculated for total lung cell number, the frequency of NP-specific T cells was increased in the lung after inoculation by the i.n. route [48]. We did not specifically measure NP-specific T cells in the lung after IDLV-NP administration. It is possible that NP-specific T cells generated in or recalled to the lung may contribute to protection.

Other mechanisms, such as the induction of mucosal IgA, may contribute to the establishment of protective immunity against influenza virus [49], [50]. We measured NP-specific IgA levels in the serum of the mice described in Fig 4B before and after 2 IDLV administrations by the i.n. or i.m. route, however these levels were quite low with no substantial differences between the two routes (data not shown). It is possible that we underestimated the levels of IgA because of partial protein degradation (the samples had been previously defrosted for measuring IgG), and we did not specifically measure NP-specific IgA in the bronchoalveolar lavages of these mice. Previous studies in IgA^−/−^ mice have suggested, however, that mucosal IgA, are not required, nor are they the primary cause for the superiority of the i.n. route of immunization [43]. A recent study further suggested that protection from influenza challenge is determined by cooperativity between influenza virus-specific T cells, non-neutralizing anti-NP antibodies, and alveolar macrophages [47]. Therefore, i.n. administration of IDLV could have the advantage of activating alveolar macrophages and other resident immune cell populations, in addition to stimulating antigen-specific cell-mediated and humoral responses. Additional studies are warranted to better characterize the immune cell subpopulations and effectors that are induced following immunization with IDLV-NP administered i.n., and to more fully elucidate the correlates of protection associated with this route.

In this proof-of-concept study, we used influenza as a model system; however, developing an influenza vaccine was not the main purpose of this study, and our initial investigation of NP expressed from IDLV need not be considered as a final choice for an influenza vaccine. In our study, we could achieve full protection from influenza mortality in mice with 2 i.n. doses of IDLV-NP, but not with a single administration of IDLV-NP by either route. Alternative strategies could improve the effectiveness of this approach. For instance, we could further diversify the immune response to our vaccine by adding another conserved influenza virus protein to IDLV-NP, such as M2. In the rAdV system, expression of NP and M2 together was more effective than either rAdV-NP or rAdV-M2 alone [43], and protection was achieved with a single dose of vaccine. Responses to IDLV may also be possibly be improved by heterologous prime/boost strategies using LVs with an envelope from a different serotype to further avoid the possibility of host anti-vector immunity [51]. Additionally, we could increase the immunogenicity of our vector by adding an adjuvant. Early studies suggested that VSV-G-pseudotyped ICLV fail to efficiently transduce fully differentiated nasal epithelium *in vivo* unless there is a disruption or modification of the epithelium barrier function [52], [53]. Although newer generations of VSV-G-pseudotyped ICLV can efficiently transduce nasal epithelial cells *in vivo* without additional treatment [54], the natural airway surfactant, lysophosphatidylcholine, when used as an adjuvant, has been shown to further enhance gene transduction [53], [55]. We did not specifically study the efficiency of airway transduction by IDLV-NP. It is therefore possible that lysophosphatidylcholine could enhance gene transduction in our system, potentially allowing for a more effective immune response.

Our study highlights the potential for IDLV to be developed for use as a novel vaccine platform. In this report, we showed that IDLV-NP are capable of inducing both cell-mediated and humoral immune responses in an antigen-specific manner. Importantly, when IDLV-NP were administered i.n., they fully protected mice from lethal challenge with homologous and heterosubtypic strains of influenza virus. Future optimization of IDLV-NP could allow for a better influenza vaccine where protection is achieved with just a single immunization. Furthermore, since IDLV can be engineered to express other antigens of interest, this vector could be adapted for use as a vaccine against other infectious diseases, and possibly cancer, making it an important tool for vaccine research and development.

## Supporting Information

Figure S1 **IDLV generate H-2k^d^-restricted T cell responses in mice.** Groups of mice (n = 3) were inoculated i.m. or i.n. 10^7^ TU/mouse of IDLV-GFP, as indicated. Splenocytes were assayed on day 10 post-immunization by ELISPOT for IFN-γ responses to an H-2k^d^-restricted 9-mer GFP peptide, or to an unrelated peptide (not shown). Mice injected with PBS served as a negative control.
(TIF)

Figure S2 **Immunization with IDLV-NP protects mice from challenge with a heterosubtypic influenza virus.** Mice (n = 5) were inoculated with IDLV-NP 2 times i.n. 4 weeks apart. Mice (n = 5) that received PBS 2 times i.n. served as a negative control, and mice (n = 5) that were infected with a sublethal dose of the mouse-adapted A/Netherlands/602/2009 (pH1N1) influenza virus served as a positive control for protection. Four weeks after the final immunization, mice were challenged with a lethal dose (10 LD_50_) of pH1N1 influenza virus and monitored for survival (**A**) and weight loss (**B**). P-values calculated using pairwise group comparisons, at each time point, were based on the ANOVA test. For weight comparisons, Day 4: p = 0.001 IDLV-NP 2X i.n. versus PBS; p = 0.000 IDLV-NP 2X i.n. versus pH1N1; Day 5: p = 0.018 IDLV-NP 2X i.n. versus PBS p = 0.003 IDLV-NP 2X i.n. versus pH1N1.; Day 6, p = 0.009 IDLV-NP 2X i.n. versus PBS; p = 0.009 IDLV-NP 2X i.n. versus pH1N1; Day 7, p = 0.002 IDLV-NP 2X i.n. versus PBS; p = 0.009 IDLV-NP 2X i.n. versus pH1N1; Day 8, p = 0.000 IDLV-NP 2X i.n. versus PBS; p = 0.011 IDLV-NP 2X i.n. versus pH1N1; Overall p-value for group comparisons based on the Kruskal-Wallis test: p = 0.002 for day 4, p = 0.004 for day 5, p = 0.003 for day 6, p = 0.005 for day 7, p = 0.009 for day 8.
(TIF)
