# Supplementary figures and images for: Mucosal Immunization with Integrase-Defective Lentiviral Vectors Protects against Influenza Virus Challenge in Mice
Source: PLoS One. 2014 May 13;9(5):e97270. doi: 10.1371/journal.pone.0097270 (PMC4019533; doi:10.1371/journal.pone.0097270)

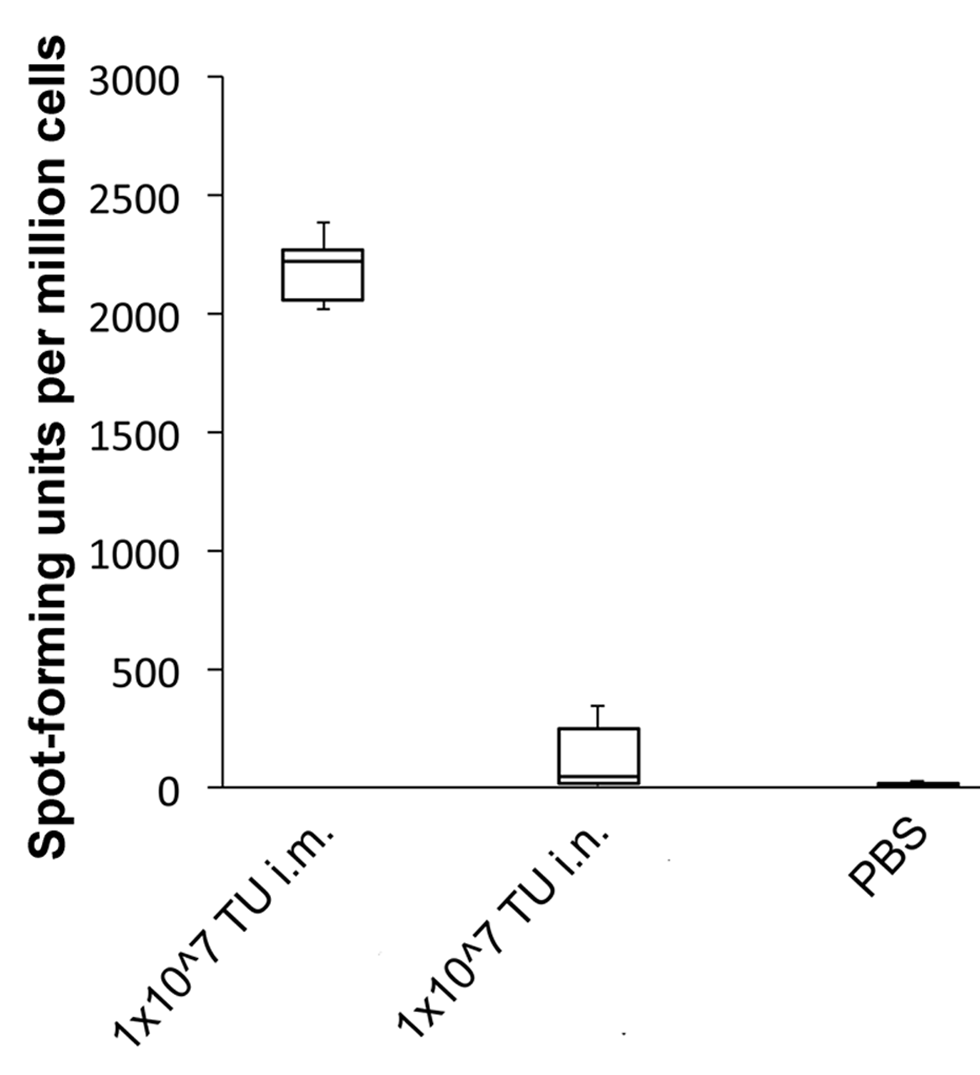

Supplement: Figure S1 — IDLV generate H-2kd-restricted T cell responses in mice. Groups of mice (n = 3) were inoculated i.m. or i.n. 107 TU/mouse of IDLV-GFP, as indicated. Splenocytes were assayed on day 10 post-immunization by ELISPOT for IFN-γ responses to an H-2kd-restricted 9-mer GFP peptide, or to an unrelated peptide (not shown). Mice injected with PBS served as a negative control. (TIF) [file pone.0097270.s001.tif]

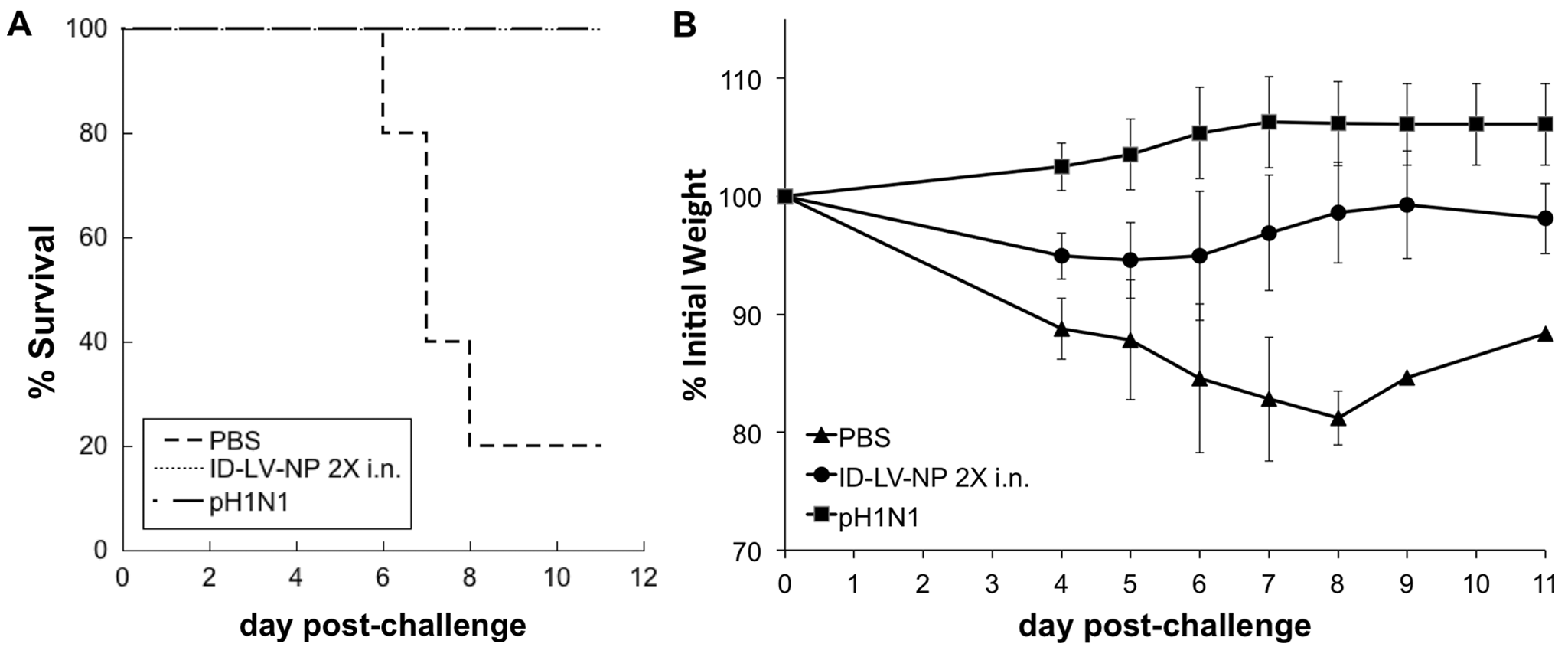

Supplement: Figure S2 — Immunization with IDLV-NP protects mice from challenge with a heterosubtypic influenza virus. Mice (n = 5) were inoculated with IDLV-NP 2 times i.n. 4 weeks apart. Mice (n = 5) that received PBS 2 times i.n. served as a negative control, and mice (n = 5) that were infected with a sublethal dose of the mouse-adapted A/Netherlands/602/2009 (pH1N1) influenza virus served as a positive control for protection. Four weeks after the final immunization, mice were challenged with a lethal dose (10 LD50) of pH1N1 influenza virus and monitored for survival (A) and weight loss (B). P-values calculated using pairwise group comparisons, at each time point, were based on the ANOVA test. For weight comparisons, Day 4: p = 0.001 IDLV-NP 2X i.n. versus PBS; p = 0.000 IDLV-NP 2X i.n. versus pH1N1; Day 5: p = 0.018 IDLV-NP 2X i.n. versus PBS p = 0.003 IDLV-NP 2X i.n. versus pH1N1.; Day 6, p = 0.009 IDLV-NP 2X i.n. versus PBS; p = 0.009 IDLV-NP 2X i.n. versus pH1N1; Day 7, p = 0.002 IDLV-NP 2X i.n. versus PBS; p = 0.009 IDLV-NP 2X i.n. versus pH1N1; Day 8, p = 0.000 IDLV-NP 2X i.n. versus PBS; p = 0.011 IDLV-NP 2X i.n. versus pH1N1; Overall p-value for group comparisons based on the Kruskal-Wallis test: p = 0.002 for day 4, p = 0.004 for day 5, p = 0.003 for day 6, p = 0.005 for day 7, p = 0.009 for day 8. (TIF) [file pone.0097270.s002.tif]
